# Mediation of lateral hypothalamus orexin input to lateral habenula in the inhibitory effects of mechanical stimulation on psychomotor responses induced by cocaine

**DOI:** 10.3389/fnmol.2023.1195939

**Published:** 2023-07-12

**Authors:** Han Byeol Jang, DanBi Ahn, Hyung Kyu Kim, Xiaowei Guan, Yu Fan, Bae Hwan Lee, Hee Young Kim

**Affiliations:** ^1^Department of Physiology, Yonsei University College of Medicine, Seoul, Republic of Korea; ^2^Department of Physiology, College of Korean Medicine, Daegu Haany University, Daegu, Republic of Korea; ^3^Department of Human Anatomy and Histoembryology, Nanjing University of Chinese Medicine, Nanjing, China

**Keywords:** orexin, mechanical stimulation, cocaine, lateral hypothalamus, lateral habenula, locomotor activity, ultrasonic vocalizations

## Abstract

**Introduction:**

The lateral hypothalamus (LH) plays an important physiological role in brain function and also plays an important role in substance abuse. The neuropeptides called orexin (or hypocretins) have been identified as being located exclusively in the cell bodies of the LH. Our previous studies have demonstrated that mechanical stimulation (MS) of the ulnar nerve produces strong inhibitory effects on cocaine addiction–like behaviors through activation of LH projection to the lateral habenula (LHb).

**Methods:**

Therefore, the present study hypothesized that ulnar MS would suppress the psychomotor responses induced by cocaine through the orexinergic LH-to-LHb pathway.

**Results:**

Ulnar MS attenuated cocaine enhancement of locomotor activity and 50-kHz ultrasonic vocalizations, which was prevented by antagonism of orexin-receptor type 2 (OX2R) in the LHb. Injection of orexin-A into the LHb reduced the cocaine-induced psychomotor responses. MS of the ulnar nerve excited LH orexinergic neurons. In addition, the excitation of LHb neurons by MS was blocked by the systemic administration of an OX2R antagonist.

**Discussion:**

These findings suggest that MS applied to the ulnar nerve recruits an orexinergic LH-to-LHb pathway to suppress the psychomotor responses induced by cocaine.

## Introduction

Recently, there has been a growing number of studies showing that the stimulation of peripheral nerves regulates the substrate of the vertebral central nervous system outside the somatosensory circuit (Bills et al., 2020; Deer et al., 2021). Our previous studies revealed that mechanical stimulation (MS) applied to peripheral sensory nerves regulates the GABA neurons of the ventral tegmental area (VTA) and dopamine (DA) release in the mesolimbic DA system, an area related to reward and motivation (Chang et al., 2017; Bills et al., 2020). In addition, our animal studies have revealed that mechanical stimulation of the ulnar nerve inhibits psychomotor activity induced by cocaine administration and these effects are mediated by the spinohypothalamic (SHT)–lateral hypothalamus (LH) pathway (Ahn et al., 2021). Furthermore, we previously proved that the lateral habenula (LHb) projects to the VTA/rostromedial tegmental nucleus (RMTg), a major GABAergic afferent to mesolimbic DA neurons, and that MS of the ulnar nerve increases the excitability of LHb neurons to suppress the mesolimbic DA system and thus inhibits cocaine-induced psychomotor responses (Chang et al., 2017).

The neuropeptide orexin-A is produced from prepro-orexin molecules secreted from hypothalamic neurons (Peyron et al., 1998). Orexin interacts with two receptors: orexin-receptor type 1 (OX1R) and orexin-receptor type 2 (OX2R). OX1R binds to orexin-A with high affinity but to orexin-B with a much lower affinity, whereas OX2R binds to both orexin peptides with high affinity (Sakurai et al., 1998). The orexin neurons in the LH regulate LHb neurons via OX2R and play an active role in reward processing and drug abuse (Harris and Aston-Jones, 2006). In addition, LHb neurons encode mRNA for both OX1R and OX2R but with a higher ratio of OX2R (Huang et al., 2019). As our previous studies showed that MS activates an LH–LHb pathway to inhibit the mesolimbic DA system and cocaine addiction–like behaviors (Chang et al., 2017; Ahn et al., 2021; Lee et al., 2022), we hypothesized that the MS effects on cocaine addiction–like behaviors may be mediated via an orexinergic pathway from the LH to the LHb.

To prove this hypothesis, the present study explored whether (1) MS applied to the ulnar nerve reduces cocaine-induced locomotor activity and 50-kHz ultrasonic vocalizations (USVs) through orexin receptors, (2) artificial increase of orexin can inhibit cocaine-induced psychomotor responses, (3) MS activates LH orexinergic neurons, and (4) activation of LHb neurons by MS is mediated by orexin.

## Materials and methods

### Animals

All experiments were performed with Sprague Dawley rats (Hyochang, Seoul, Korea), weighing 240–400 g. All the tasks were performed using male rats unless stated otherwise. The animals were housed under a constant temperature (25 ± 2°C) and a 12-h light–dark cycle with free access to water and food. All procedures were approved by the Institutional Animal Care and Use Committee (IACUC) at Yonsei University and Daegu Haany University and conducted according to the National Institutes of Health Guide for Care and Use of Laboratory Animals.

### Chemicals and reagents

Cocaine hydrochloride (15 mg/kg in saline, Macfarlan Smith Ltd., Edinburgh, United Kingdom), orexin-A [333 pmol/μL in artificial cerebrospinal fluid (aCSF); Tocris Bioscience, United Kingdom], orexin-B [333 pmol/μL in artificial cerebrospinal fluid (aCSF); 10 ug/loci; Tocris Bioscience, United Kingdom] (Zhou et al., 2023), and TCS-OX2-29 (an OX2R antagonist; 33.3 μg/μL in 5% DMSO aCSF; Tocris Bioscience, United Kingdom) were used. The brain injection was carried out with a micro-pump (pump 22, Harvard Apparatus, Holliston, MA, United States) at a speed of 0.25 μL/min for 5 min on each side.

### Cannula implantation

Under sodium pentobarbital anesthesia (50 mg/kg, i.p.), double-barreled guide cannulas (26-gauge, double 1.6-mm C-C) were implanted bilaterally into the LHb (anterior: −3.8 mm; lateral: ±0.8 mm; deep: −4.8 mm) to locally infuse the orexin-A or -B or orexin antagonist TCS-OX2-29. The experiment was performed after recovery of at least 7 days.

### MS of the ulnar nerve

MS was carried out by using a mechanical instrument (MI) as performed in our previous study. In brief, for MS of the ulnar nerve, needles (0.18 mm in diameter, 8 mm in length, Dong Bang Medical Co., Qingdao, China) were inserted bilaterally 3-mm deep into the ulnar tunnel on the transverse crease of the wrist of the forepaw. MI consisted of a custom-made control unit and a cell phone vibrator (MB-0412 V, Motor bank, Korea) mated to a needle. Prior to experiment, intensity of mechanical stimulation by MI was measured as described previously (Kim et al., 2013). In brief, a tip of needle was attached to an accelerometer (PO-AXA-12-01, Intellane Co., Korea) which converted acceleration (intensity) from motion to voltage. The signals from the accelerometer were digitized and analyzed using a data acquisition card (DAQ-NI USB-6200, National Instruments, Austin, TX, United States) and a customized LabVIEW (National Instruments, Austin, TX, United States) virtual instrument software program. By using our MI, the inserted needle was mechanically stimulated for 20 s in duration at an intensity of 1.3 m/s^2^, left without mechanical stimulation for further 40 s and subsequently withdrawn.

### Measurement of locomotor activity and 50-kHz USVs

As performed in our laboratory, locomotor activity and 50-kHz USVs were recorded simultaneously in customized sound-attenuating chambers (Kim et al., 2021; Lee et al., 2022). The chamber consisted of two boxes to minimize exterior noise (inside box: 54 × 38 × 35 cm, outside box: 68 × 50 × 51 cm). A condenser ultrasonic microphone (Ultramic250K; Dodotronic, Castel Gandolfo, Italy) and a digital camera were positioned at the center of the ceiling of the chamber. The 50-kHz USVs were recorded using the ultrasonic microphone with an UltraSoundGate 416H data acquisition device (Avisoft Bioacoustics, Glienicke, Germany). Ultrasonic vocal signals were band-filtered between 38 and 96 kHz for the 50-kHz USVs and analyzed using Avisoft-SASLab Pro (version 4.2; Avisoft Bioacoustics, Glienicke, Germany). Locomotor activity was measured with a video-tracking system (Ethovision XT; Noldus Information Technology BV, Wageninge, Netherlands). After recording baseline activity for 30 min, the rats were given an intraperitoneal injection of cocaine (15 mg/kg) and/or MS and monitored for up to 60 min after cocaine injection. The data were expressed as the distance traveled for locomotor behaviors and the numbers of 50-kHz USVs during each 10 min period.

### *In vivo* extracellular single-unit recording of LH and LHb neurons

Single-unit discharges from LH and LHb neurons were recorded as described in our previous publications (Chang et al., 2017; Ahn et al., 2021). Briefly, under urethane anesthesia (1.5 g/kg, i.p.), rats were positioned on a stereotaxic apparatus and small holes were drilled into the skull to accommodate recording electrodes. A single carbon-filament glass microelectrode (Carbostar-1, impedance 0.4 ~ 1.2 MΩ, Kation Scientific, Minneapolis, MN, United States) was stereotaxically positioned in the LH areas (AP −2.5 mm, ML ±1.4 mm, DV −8.8 mm from the skull) and LHb areas (AP −3.6 ~ −3.8 mm, ML ±0.8 ~ ±0.9 mm, DV −4.8 ~ −5.4 mm from the skull) (Paxinos and Watson, 2006). For recording of LH neurons, a stable baseline of at least 10 min was recorded, and the unit activity following MS was further recorded for 10 min. For recording of LHb neurons, after recording basal activity for at least 10 min, an OX2R antagonist TCS-OX2-29 was injected intraperitoneally, and MS was applied 10 min after the injection.

### Response of LH orexinergic neurons to MS and identification of LH-LHb projection

Immunohistochemical analysis of c-Fos and orexin-A in LH following MS was carried out as described previously (ref). In brief, 30 min after bilateral ulnar MS, the brains were removed, post-fixed in 4% paraformaldehyde and cryoprotected in 30% sucrose. The tissue was then cryosectioned into 30 μm-thick. The tissues were incubated with mouse anti-c-Fos antibody (1:500, Santa Cruz Biotechnology) and rabbit anti-orexin-A peptide (1:1000, Abcam), followed by incubation with secondary antibodies (1:200, Alexa Fluor 488 donkey anti-rabbit IgG antibody, Thermo Scientific, MA, United States; 1:200, Alexa Fluor® 594 donkey anti-mouse IgG antibody, Thermo Scientific). The slides were washed and cover-slipped with Vectashield Hard Set mounting medium. All samples were taken from 3 to 5 sections from each animal. All images were taken with a fluorescence microscope (BX51; Olympus, Hamburg, Germany), and positive cells were blindly counted.

To identify LH-LHb projection, viral vectors were injected as described previously (Ahn et al., 2021). Under pentobarbital anesthesia (50 mg/kg, i.p.), AAV5-hSyn-EYFP (0.5 μL/loci; 7 × 10^12^ vg/mL, Addgene) was stereotaxically injected into the bilateral LH (stereotaxic coordinates: posterior, −2.5 mm; lateral, ±1.8 mm; ventral, 8.8 mm) over 10 min with an additional 6 min for diffusion. Three weeks after viral injection, brains were removed, post-fixed in 4% paraformaldehyde and cryoprotected in 30% sucrose. The tissue was then cryosectioned into 30 μm-thick and examined under a confocal laser scanning microscope (LSM700, Carl Zeiss, Germany).

### Statistical analysis

Data were presented as the mean ± standard error of the mean and analyzed by one- or two-way repeated measurement (RM) analysis of variance (ANOVA), followed by *post hoc* testing using the Tukey method, where appropriate. Values of *p* less than 0.05 were regarded as statistically significant.

## Results

### Effect of an OX2R antagonist injection into the LHb on the inhibition by ulnar MS of cocaine-induced 50-kHz USVs and locomotor activity

**Figure 1:**
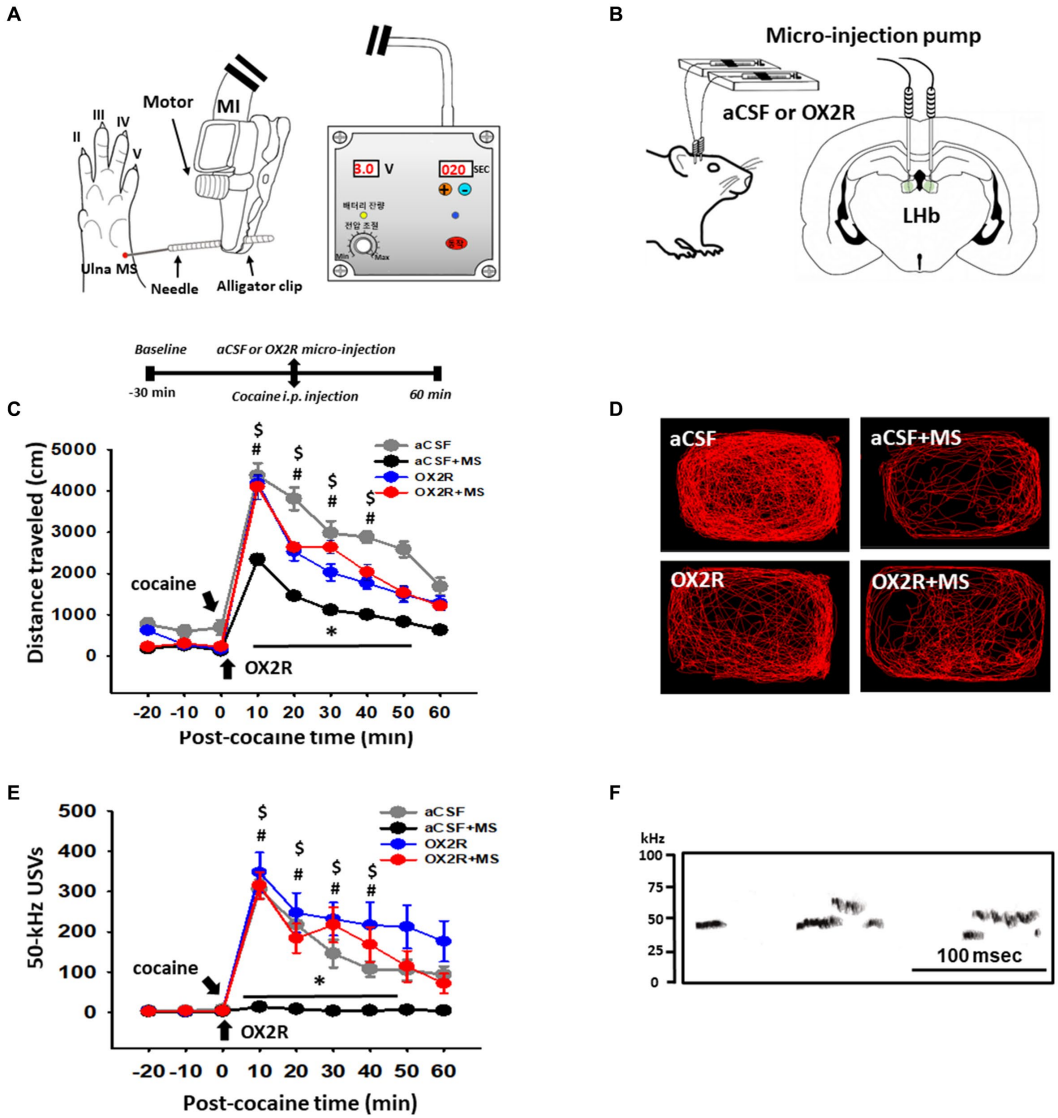
Effect of injection of orexin-receptor type 2 antagonist (OX2R) TCS-OX2-29 into the lateral habenula (LHb) on mechanical stimulation (MS)-induced inhibition of cocaine-enhanced locomotion and 50-kHz ultrasonic vocalizations (USVs). **(A)** Experimental schedule and schematic location of MS sites. The MS sites were stimulated with a mechanical instrument (MI). **(B)** Schematic for infusion of an OX2R antagonist TCS-OX2-29 into the LHb of rats. **(C)** Effect of an OX2R antagonist TCS-OX2-29 on MS-induced inhibition of cocaine-enhanced locomotion in rats. *p < 0.001, aCSF vs. aCSF+MS; #*p* < 0.05, OX2R vs. aCSF+MS; ^$^*p* < 0.05 OX2R + MS vs. aCSF+MS. **(D)** Representative moving tracks for 60 min after cocaine in each group (*n* = 6 per group). **(E)** Effect of TCS-OX2-29 on MS-induced inhibition of cocaine-enhanced 50-kHz USVs in rats. *p < 0.001, aCSF vs. aCSF+MS; #*p* < 0.05, OX2R vs. aCSF+MS; ^$^*p* < 0.05 OX2R vs. aCSF+MS. **(F)** Representative spectrograms of 50-kHz USVs following cocaine injection. Spectrograms display 50-kHz USVs elicited about 10 min after cocaine injection (15 mg/kg, i.p.) in a representative rat (OX2R group).

To test whether MS reduces cocaine locomotion via orexin, an orexin-receptor type 2 (OX2R) antagonist was injected into the LHb prior to cocaine administration and MS (Figures 1A,B). Systemic injection of cocaine rapidly increased locomotor activity and 50-kHz USVs, with a peak at 10 min, compared with the values before cocaine injection (Figures 1C,E). MS significantly reduced both cocaine-induced locomotor activity and 50-kHz USVs, compared with a control group [*p* < 0.001, aCSF vs. aCSF+MS; locomotion: two-way RM ANOVA, Tukey’s test, treatment, *F*_(3,168)_ = 13.425, *p* < 0.001; time, *F*_(8,168)_ = 115.232, *p* < 0.001; interaction, *F*_(24.168)_ = 2.617, *p* < 0.001; USVs: two-way RM ANOVA, treatment, Tukey’s test, *F*_(3,120)_ = 34.596, *p* < 0.001; time, *F*_(8,120)_ = 124.413, *p* < 0.001; interaction, *F*_(24,120)_ = 3.808, *p* < 0.001; Figures 1C,E]. In another set of animals, we investigated sex differences in the effects of ulnar MS on cocaine-induced psychomotor responses. Ulnar MS attenuated cocaine-induced locomotion in both males (*n* = 6) and females (*n* = 6). Although male rats seemed to be superior to female rats in suppression of cocaine locomotion by ulnar MS, there is no significant difference in ulnar MS’s effect between males and females (Supplementary Figure S1).

The inhibitory effects of MS on cocaine-induced locomotion and 50-kHz USVs in male rats were blocked when an OX2R antagonist TCS-OX2-29 was injected into the LHb prior to cocaine injection and MS. On the other hand, the OX2R antagonist itself did not affect locomotor activity and 50-kHz USVs (*p* > 0.05, OX2R vs. aCSF; Figures 1C–F).

### Effect of orexin injection into the LHb on cocaine-induced 50-kHz USVs and locomotor activity

**Figure 2:**
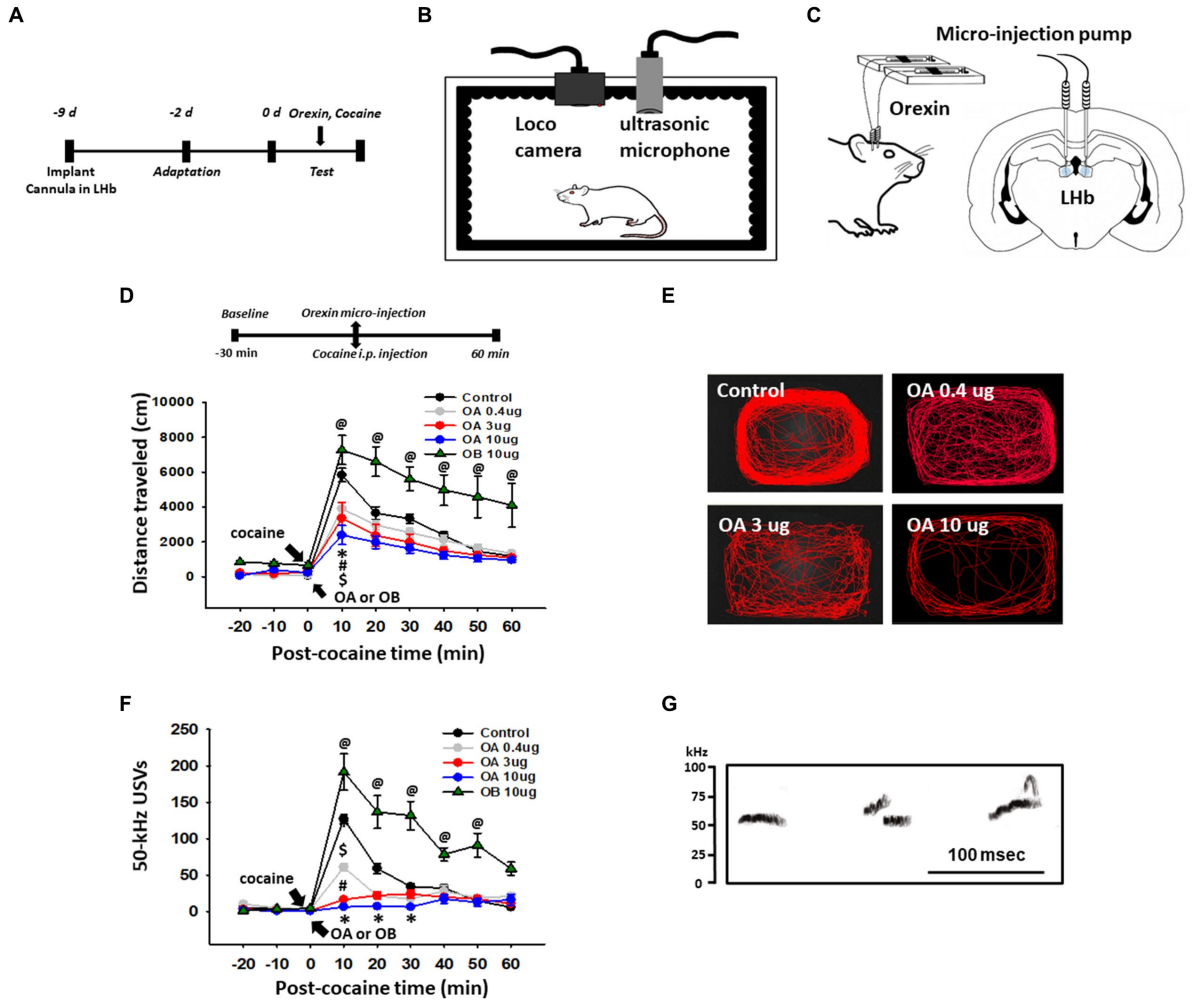
Effect of infusion of orexin-A in the lateral habenula (LHb) on cocaine-enhanced locomotion and 50-kHz ultrasonic vocalizations (USVs). **(A)** Experimental schedule. **(B)** Schematics for locomotion and USVs recordings in a sound-attenuating chamber. **(C)** Schematic for the orexin infusion into the LHb of rats. **(D)** Effect of infusion of orexin-A (OA) or orexin-B (OB) into the LHb on cocaine-induced locomotor activity in rats. ^*^p < 0.001, Control vs. OA 10 μg; ^#^*p* < 0.001, Control vs. OA 3 μg; ^$^*p* < 0.001, Control vs. OA 10 μg; ^@^Control vs. OB 10 μg. **(E)** Representative moving tracks for 60 min after cocaine injection in each group. **(F)** Effect of infusion of orexin-A or orexin-B into the LHb on cocaine-induced 50-kHz USVs in rats. ^*^p < 0.05, Control vs. OA 10 μg; ^#^*p* < 0.001, Control vs. OA 3 μg; ^$^*p* < 0.001, Control vs. OA 0.4 μg; ^@^*p* < 0.001, Control vs. OB 10 μg (*n* = 6 per group). **(G)** Representative spectrograms of 50-kHz USVs following cocaine injection. Spectrograms display 50-kHz USVs elicited about 10 min after cocaine injection (15 mg/kg, i.p.) in a representative rat (Control group).

To test whether orexin activation of the LHb can mimic the MS effects, guide cannulas were implanted bilaterally into the LHb and the behavioral experiment was performed 7 days after the surgery (Figures 2A–C). Systemic cocaine increased locomotor activity and 50-kHz USVs (Control, aCSF injection into LHb; Figures 2D–G). When various concentrations of orexin-A (OA) were injected bilaterally into the LHb prior to cocaine injection, the cocaine-enhanced locomotor activity and 50-kHz USVs were attenuated in a dose-dependent manner [Locomotion: two-way ANOVA, Tukey’s test, treatment, *F*_(4,2070)_ = 40.705, *p* < 0.001; time, *F*_(8,207)_ = 51.370, *p* < 0.001; interaction, *F*_(32,120)_ = 2.299, *p <* 0.001; USVs: two-way RM ANOVA, Tukey’s test, treatment, *F*_(4,96)_ = 34.604, *p* < 0.001; time, *F*_(8,96)_ = 57.511, *p* < 0.001; interaction, *F*_(32,96)_ = 11.512, *p* < 0.001; Figures 2D,F]. In contrast, injection of orexin-B (OB) into LHb enhanced the cocaine-induced locomotion and 50-kHz USVs, compared to control (*p* < 0.05 vs. Control).

### Activation of LH neurons by MS in rats

**Figure 3:**
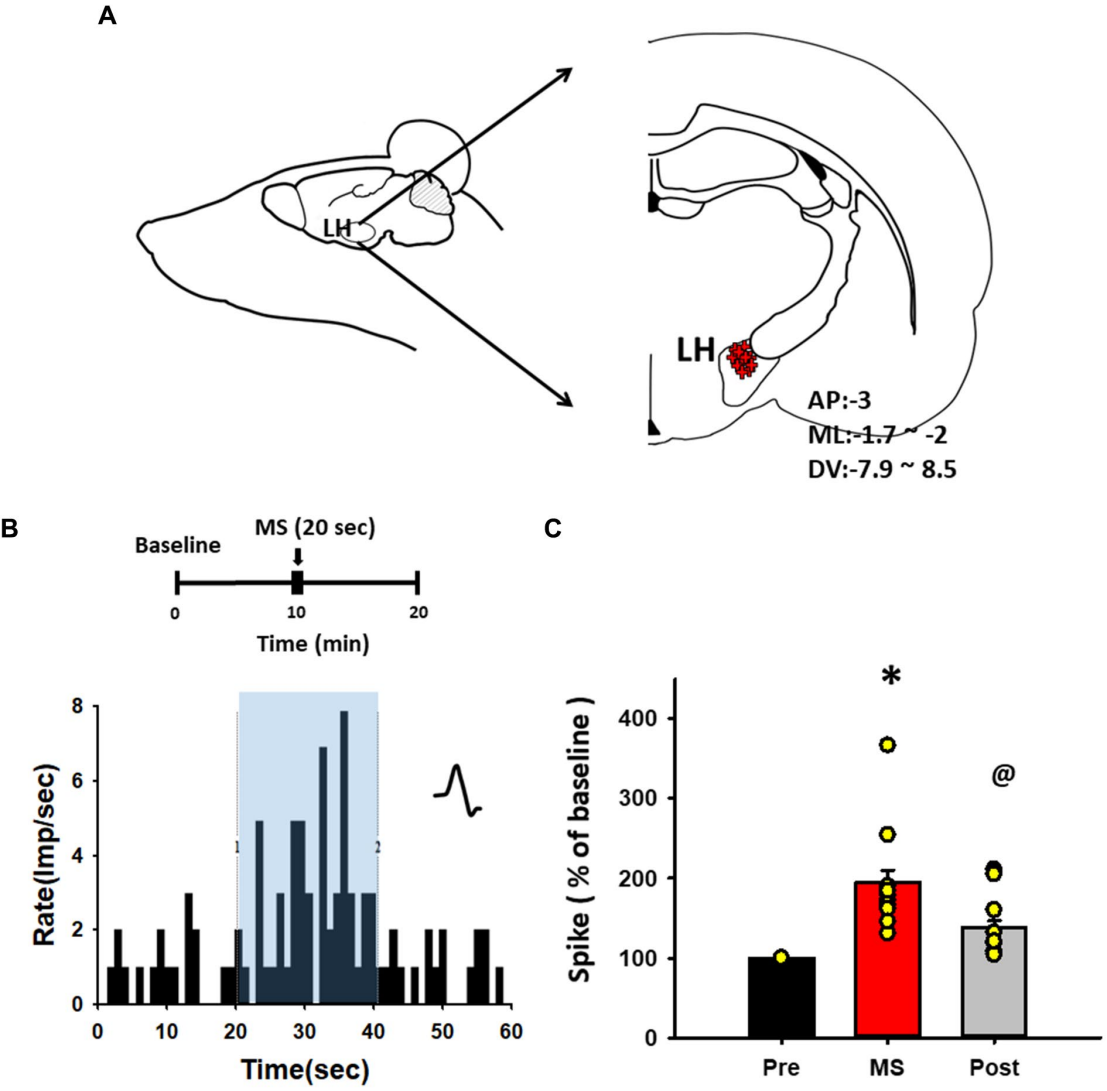
*In vivo* extracellular single-unit recordings of lateral hypothalamus (LH) neurons following mechanical stimulation (MS). **(A)** Schematic and coordinate values for *in vivo* extracellular single-unit recordings of LH neurons. **(B)** Representative histogram for single-unit discharges from LH neurons before, during and after MS. **(C)** Mean spike frequency from baseline before (Pre, 20 s), during (MS, 20 s) and after (Post, 20 s) MS. Single-unit activities of LH neurons were evoked during MS in rats. *p < 0.001, Pre vs. MS, ^@^*p* < 0.05, MS vs. Post (*n* = 10 per group).

To determine whether MS excites LH neurons, *in-vivo* extracellular single-unit recordings were performed in LH neurons during MS of the ulnar nerve. After a stable baseline for at least 10 min was established, the neuronal activity following MS was recorded for another 10 min (Figures 3A,B). MS increased the firing rate of LH neurons by 195.16% ± 14.94% from baseline during stimulation [one-way RM ANOVA, *F*_(2,18)_ = 15.168, *p* < 0.001; Figure 3C] and single-unit discharges for 20 s after MS were 138.46% ± 8.3% over baseline [one-way RM ANOVA, F_(2,18)_ = 15.168, *p* < 0.05; Figure 3C].

### Response of LHb neurons to MS in acute OX2R antagonist-treated rats

**Figure 4:**
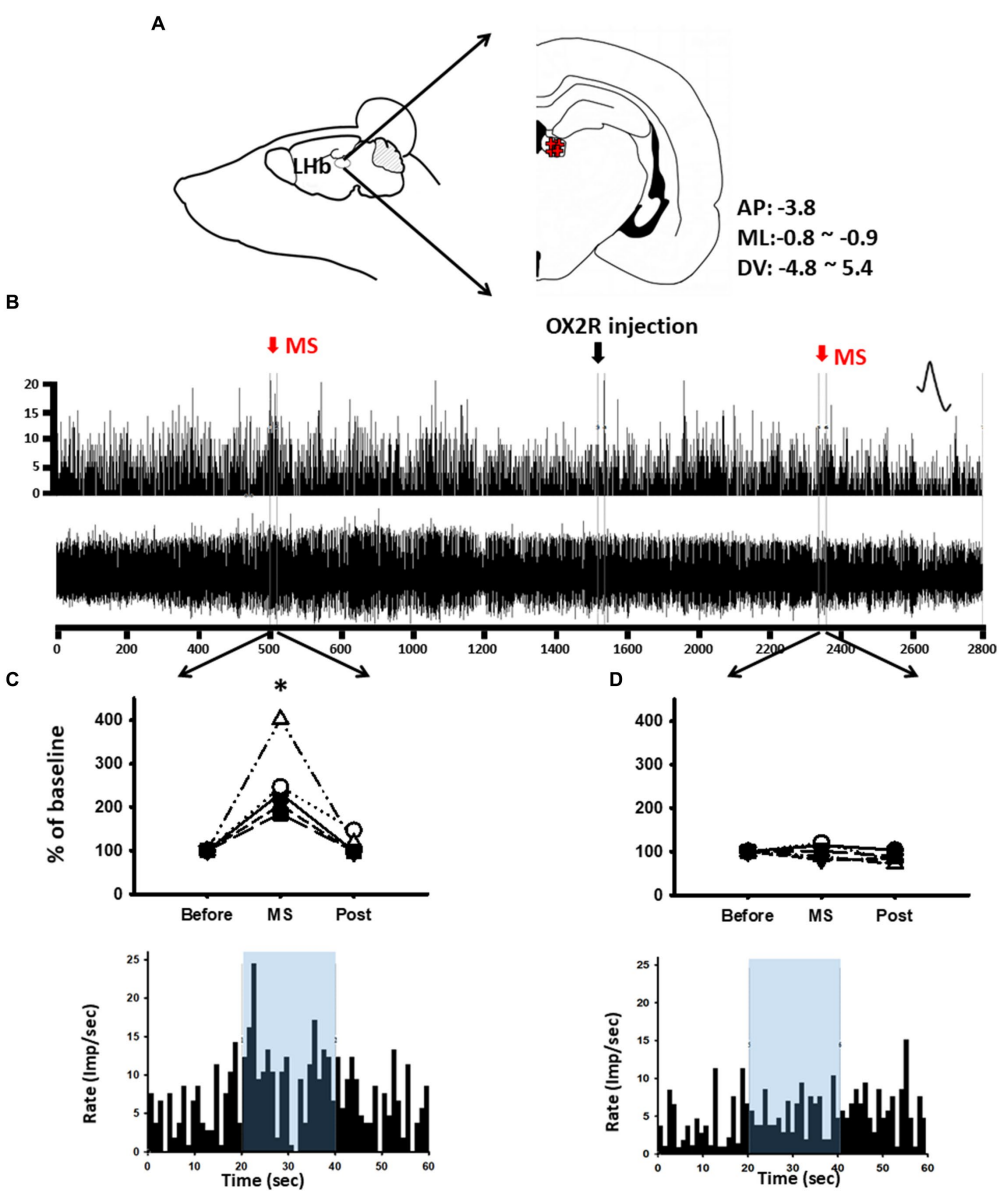
Blockade of mechanical stimulation (MS)-induced lateral habenula (LHb) neuronal activity by OX2R antagonist TCS-OX2-29. **(A)** Schematics and coordinate value for *in-vivo* extracellular single-unit recordings of LHb neurons. **(B)** Single-unit discharges from LHb neurons for 50 min. **(C)** Mean spike frequency/s before and after MS. *p < 0.001 (*n* = 5). **(D)** Mean spike frequency/s before and after OX2R and/or MS. Increased single-unit activities of LHb neurons following MS were inhibited after systemic injection of an OX2R antagonist TCS-OX2-29.

To determine whether the increased activity of LHb neurons following MS was associated with orexin, *in-vivo* extracellular single-unit recordings of LHb neurons were carried out in the rats given MS and systemic administration of an OX2R antagonist, TCS-OX2-29. When a baseline of stable LHb neurons for at least 10 min was established, vehicle or OX2R antagonist was systemically injected. Ten minutes after the injection, MS was performed for 20 s, and the single-unit discharges were then measured (Figures 4A,B). Single-unit discharge rates of LHb neurons increased by 254.21% ± 31.42% in response to MS compared with baseline [one-way RM ANOVA, *F*_(2,8)_ = 15.306, *p* < 0.01, Before (20 s before MS) vs. MS (20 s during MS); Figure 4C]. On the other hand, when MS after systemic injection of TCS-OX2-29 was applied, excitation of LHb neurons following MS was almost completely blocked (Figure 4D).

### Excitation of LH orexinergic neurons by MS and LH-LHb projection

**Figure 5:**
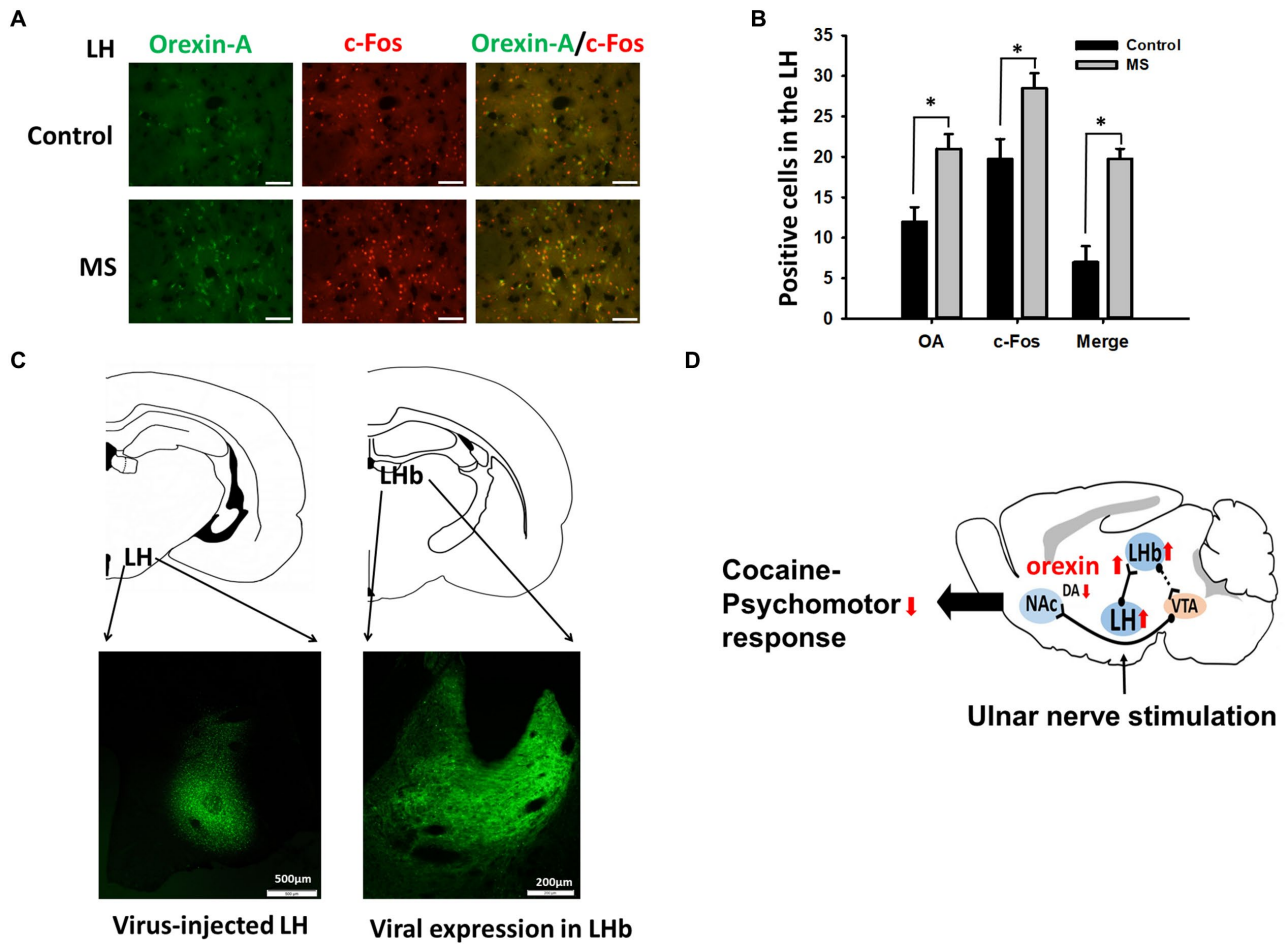
Excitation of LH Orexinergic Neurons by MS and LH-LHb projection. **(A,B)** Immunohistochemistry for orexin-A and c-Fos in MS-treated or control rats. Mean numbers of orexin-A (OA) and/or c-Fos positive cells in the LH of control (no MS; *n* = 5) or MS-treated rats (*n* = 5), **(B)** Scale bar = 50 μm. **(C)** Expression of AAV5-hSyn-EYFP in LHb 3 weeks after injection into LH. **(D)** A summary diagram of this study. Mechanical stimulation applied to the ulnar nerve activates the orexin-operated projection from the LH to the LHb, which excites VTA GABA neurons to inhibit VTA DA neurons, resulting in suppression of the psychomotor responses induced by cocaine.

To see whether LH orexinergic neurons can be excited by MS, the animals were sacrificed 30 min after MS and double immunohistochemical staining for orexin-A and c-Fos was performed in the LH of MS-stimulated rats (*n* = 5) or control rats (*n* = 5). MS significantly increased both orexin-A and c-Fos expressions, compared to control. Significantly increased number of double-stained cells for c-Fos and orexin-A was found in MS-treated rats, compared to control (Figures 5A,B). When AAV5-hSyn-EYFP was injected into LH, high expression of virus was observed in the LHb (Figures 5C,D), indicating LH-LHb projection.

## Discussion

In the present study, MS applied to the ulnar nerve attenuated cocaine enhancement of locomotor activity and 50-kHz USVs, which was blocked by injection of an OX2R antagonist into the LHb. Injection of orexin-A, but not orexin-B, into the LHb reduced the cocaine-induced locomotor activity and 50-KHz USVs. MS of the ulnar nerve excited the LH neurons. In addition, MS excited the LHb neurons, which was inhibited by systemic administration of an OX2R antagonist. Immunohistochemistry showed that MS activated LH orexinergic neurons. These findings suggest that MS applied to the ulnar nerve activates the orexin-operated projection from the LH to the LHb, thereby leading to attenuation of the psychomotor responses induced by cocaine.

While the field of pharmacological addiction treatment is expanding rapidly, there is also an increasing interest in the use of non-pharmacological interventions in treatment of drug addiction (Carroll and Schottenfeld, 1997). As a non-pharmacological therapy, our previous studies have shown that somatosensory stimulation such as ulnar MS or stimulation of HT7 acupoints over ulnar nerve suppresses drug-induced psychomotor responses or self-administration behavior or relapse to abused drugs such as cocaine, morphine and ethanol (Lee et al., 2014; Chang et al., 2017, 2019; Jang et al., 2023). We also found that MS of the ulnar nerve, but not the radial nerve, attenuates cocaine-induced psychomotor responses through activation of A-fiber at the nerve trunk, the dorsal column–medial lemniscus pathway, and LHb neurons (Kim et al., 2013; Chang et al., 2017). The afferent inputs generated from ulnar MS reduce drug-induced DA release in the nucleus accumbens (NAc) by modulating VTA GABA neurons in rats (Yang et al., 2010; Chang et al., 2017; Bills et al., 2020). Our previous study showed that stimulation of bilateral radial nerves did not affect cocaine-induced locomotion (Kim et al., 2013; Chang et al., 2017). Indeed, it was reported that unilateral stimulation of the radial nerve at low thresholds produces the opposite effects on DA activity in anesthetized cats, characterized by a DA decrease in the contralateral midbrain and a DA increase in the ipsilateral midbrain (Nieoullon and Dusticier, 1982). When bilaterally stimulated, the radial nerve did not have a significant effect on midbrain DA activity or cocaine-induced psychomotor behaviors. Thus, it suggests that MS can reduce drug addiction behavior in a nerve-dependent manner. In the present study, ulnar MS excited LH and LHb neurons and reduced 50-kHz USVs, known to be associated with NAc DA release (Chang et al., 2017; Ahn et al., 2021; Lee et al., 2022). The LH projects to LHb neurons that, in turn, directly innervate RMTg GABA neurons and indirectly inhibit DA neurons (Lazaridis et al., 2019). Our previous studies revealed that ulnar MS activates RMTg GABA neurons and suppresses the mesolimbic DA system (Chang et al., 2017; Ahn et al., 2021). Thus, ulnar MS may excite the LH neurons projecting to LHb, which would, in turn, activate RMTg GABA neurons and inhibit VTA DA neurons, leading to the suppression of cocaine-induced psychomotor behaviors.

In the present study, infusion of orexin-A, but not orexin-B, peptides into the LHb suppressed cocaine-enhanced locomotion and 50-kHz USVs in a dose-dependent manner. Indeed, the role of orexin signaling from the LH-to-LHb glutamatergic neurons in addiction remains unexplored. Recent studies reported that orexinergic projections from the LH to the LHb modulate aggressive behaviors and social defeat behaviors (Flanigan et al., 2020). Wang et al. showed that in socially defeated mice, orexinergic neurons in the LH and glutamatergic neurons in the LHb are strongly activated (Wang et al., 2021) and a large population of OX2R-expressing neurons in the LHb is glutamatergic. An increase in orexin-A peptides enhanced the synaptic activity and spike frequency by presynaptic promotion of glutamate release in neuronal cells (Li et al., 2002; Jeon et al., 2015). In the present study, cocaine-induced psychomotor responses were suppressed by MS of the ulnar nerve, which was prevented by injection of OX2R antagonist TCS-OX2-29 into the LHb. Furthermore, infusion of orexin-A peptides, but not orexin-B, into the LHb suppressed cocaine-induced psychomotor responses. Excitation of the LHb by ulnar MS was blocked by antagonizing OX2R. In addition, glutamatergic LHb neurons directly innervate RMTg GABA neurons and indirectly inhibit VTA DA neurons (Lazaridis et al., 2019). The present study also showed that MS activated LH orexinergic neurons. These results suggest that ulnar MS activates orexinergic LH neurons projecting to the LHb and that the elevated orexin-A peptides increase glutamatergic transmission of the LHb, which activates RMTg GABA neurons and inhibits VTA DA neurons, resulting in suppression of the psychomotor responses induced by cocaine.

The LHb is a critical node within the reward circuitry of humans and nonhuman animals that, when activated, promotes negative emotional states predominantly through indirect inhibition of midbrain DA neurons (Matsumoto and Hikosaka, 2007; Baker et al., 2016). Optogenetic inhibition of mouse LHb neurons during the test phase of the aggression conditioned place preference (CPP) task increases the time spent in the aggression-paired context, whereas optogenetic activation of these neurons reduces it (Golden et al., 2016). Optogenetic activation of orexin inputs to the LHb increases aggressive behavior. These studies suggest that ulnar MS recruits an orexinergic LH-to-LHb pathway to suppress psychomotor responses induced by cocaine administration.

In conclusion, mechanical stimulation applied to the ulnar nerve activates the orexin-operated projection from the LH to the LHb and attenuates the psychomotor responses induced by cocaine.

## Data availability statement

The original contributions presented in the study are included in the article/Supplementary material, further inquiries can be directed to the corresponding author.

## Ethics statement

The animal study was reviewed and approved by Yonsei University and Daegu Haany University.

## Author contributions

HYK conceived and designed the research. HJ, DA, and HKK performed the research. HJ, BL, XG, and YF analyzed the data and drafted the manuscript. HYK was responsible for the overall direction of the project and editing the manuscript. All authors contributed to the article and approved the submitted version.

## Funding

This research was supported by a National Research Foundation of Korea (NRF) grant funded by the Korean Government (MSIT) (2019R1A2C100255514), the Korea Institute of Oriental Medicine (KIOM) (KSN1812181, KSN2013210), and the Korea Institute of Planning and Evaluation for Technology in Food, Agriculture and Forestry (IPET) through the Companion Animal Life Cycle Industry Technology Development Program, which is funded by the Ministry of Agriculture, Food and Rural Affairs (MAFRA) (322096–5).

## Conflict of interest

The authors declare that the research was conducted in the absence of any commercial or financial relationships that could be construed as a potential conflict of interest.

## Publisher’s note

All claims expressed in this article are solely those of the authors and do not necessarily represent those of their affiliated organizations, or those of the publisher, the editors and the reviewers. Any product that may be evaluated in this article, or claim that may be made by its manufacturer, is not guaranteed or endorsed by the publisher.

## Supplementary material

SUPPLEMENTARY FIGURE 1 Effect of ulnar MS on cocaine-induced locomotor activity in male and female rats. All groups received aCSF injection into LHb prior to cocaine administration. **p* < 0.05 vs. Control. Control (M) = naïve male rats; MS (M) = ulnar MS in male rats; Control (F) = naïve female rats; MS (F) = ulnar MS in female rats. There is no significant difference in the inhibitory effects of MS on cocaine-induced locomotion between males and females (*p* > 0.05, MS (M) vs. MS (F)).
