# Supplementary figures and images for: Mediation of lateral hypothalamus orexin input to lateral habenula in the inhibitory effects of mechanical stimulation on psychomotor responses induced by cocaine
Source: Front Mol Neurosci. 2023 Jul 12;16:1195939. doi: 10.3389/fnmol.2023.1195939 (PMC10369078; doi:10.3389/fnmol.2023.1195939)

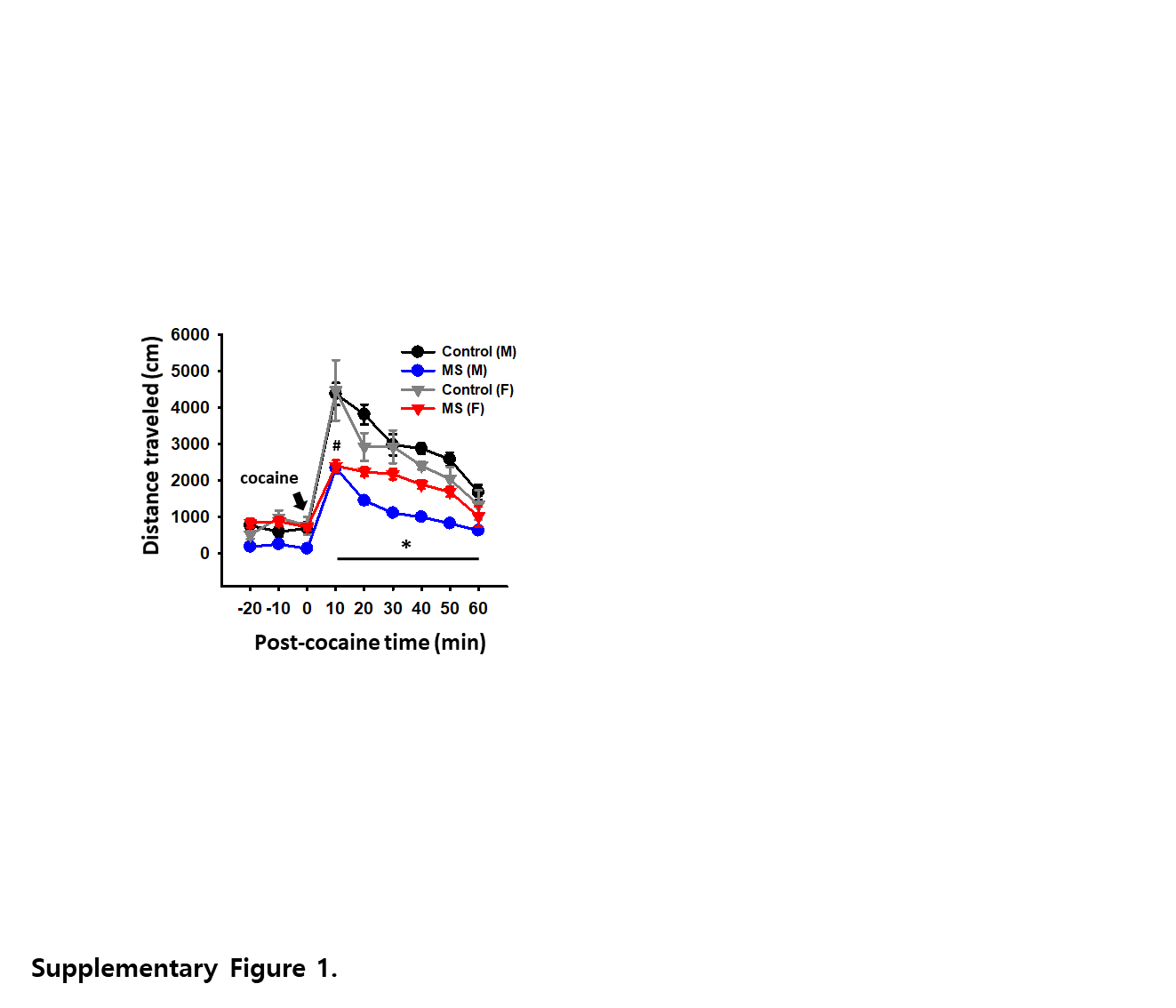

Supplement: SUPPLEMENTARY FIGURE 1 — Effect of ulnar MS on cocaine-induced locomotor activity in male and female rats. All groups received aCSF injection into LHb prior to cocaine administration. *p < 0.05 vs. Control. Control (M) = naïve male rats; MS (M) = ulnar MS in male rats; Control (F) = naïve female rats; MS (F) = ulnar MS in female rats. There is no significant difference in the inhibitory effects of MS on cocaine-induced locomotion between males and females (p > 0.05, MS (M) vs. MS (F)). [file Image_1.TIF]
